# Multifaceted CK2 in malignant and healthy T cells

**DOI:** 10.18632/oncotarget.21700

**Published:** 2017-10-09

**Authors:** Sérgio T. Ribeiro, João T. Barata, Bruno Silva-Santos

**Affiliations:** Bruno Silva-Santos: Instituto de Medicina Molecular, Faculdade de Medicina, Universidade de Lisboa, Lisboa, Portugal; João T. Barata: Instituto de Medicina Molecular, Faculdade de Medicina, Universidade de Lisboa, Lisboa, Portugal

**Keywords:** protein kinase CK2, γδ T lymphocytes, T-cell acute lymphoblastic leukemia

Among kinases that support the survival and turnover of tumor cells, the serine/threonine protein kinase CK2 has been shown to be frequently overexpressed or hyperactivated in solid and hematological malignancies. Our previous work on T-cell acute lymphoblastic leukemia (T-ALL) showed that CK2 maintains leukemia cell viability by phosphorylating and thereby inactivating the tumor suppressor PTEN, which results in hyperactivation of PI3K/AKT signaling [1]. We also demonstrated the potential of using the clinical-grade CK2-specific chemical inhibitor, CX-4945 (Silmitasertib), against primary T-ALL cells [2]. We have now [3] extended these findings to the rare (<10% of all cases) form of T-ALL that derives from the transformation of thymocytes belonging to the γδ T-cell lineage [4].

γδ T cells develop in the thymus from progenitors common to the αβ T-cell lineage, and constitute a small population (1-5%) of the circulating T lymphocyte pool of healthy humans. In leukemia, malignant γδ T cells cause the γδ T-ALL subtype that presents with distinctive clinical features [4]. However, the molecular mechanisms that support γδ T-ALL homeostasis and progression remain poorly understood.

In our most recent study, we identify CK2 as a novel determinant of γδ T-ALL cell survival [3]. Consistent with previous T-ALL data [1-2], CK2 promoted γδ T-ALL cell survival partly via AKT signaling, as demonstrated by the negative effects of CX-4945 on PTEN and AKT phosphorylation; the impact of the AKT inhibitor MK-2206; and the partial rescue of CX-4945-induced apoptosis upon ectopic expression of a constitutively active AKT isoform [3]. Remarkably, however, primary γδ T-ALL cells were clearly more dependent on CK2 than αβ T-ALL cells, with significantly higher CK2 activity and sensitivity to CX-4945. Accordingly, CX-4945 treatment inhibited tumor progression and dissemination in a xenograft model of γδ T-ALL, thus supporting CK2 inhibition as a therapeutic approach in γδ T-ALL.

Interestingly, our study also revealed an unanticipated role for CK2 in controlling the survival of normal γδ thymocytes [3], which contrasted with our previous findings on healthy αβ thymocytes [1]. Thus, γδ thymocytes presented increased CK2 activity (which was further upregulated upon TCR stimulation) than their αβ counterparts; and, unlike these, were highly susceptible to CX-4945-induced apoptosis. This suggests that a possible unwanted side effect of CX-4945 treatment in cancer patients may be the depletion of healthy γδ T cells that provide anti-tumor immune surveillance [5].

Of note, studies in mice have suggested that CK2 is also involved in the suppressive function of CD4^+^ Foxp3^+^ regulatory T cells against allergy-promoting Th2 cells [6]; and in naïve CD4^+^ T cell differentiation into Th2 or Th17 cells [7]. Thus, CK2 inhibition may impact on other protective (against infection) or pathogenic (allergic or auto-inflammatory) immune responses.

The mechanisms of regulation of CK2 activity remain largely unknown, with very few studies demonstrating the modulation of CK2 activity by extracellular factors. Our results show that CK2 activity can be enhanced by TCR and CD27 (co)stimulation in γδ T cells [3]. In a different study, we have also demonstrated that IL-7/IL-7R stimulation upregulates CK2 activity, which is required for maximal IL-7R-triggered JAK1/STAT5 and AKT signaling, and consequent pro-leukemia effects, in T-ALL cells [8]. Therefore, major molecular cues in T cell development may converge in increasing (by yet unclear mechanisms) CK2 activity in healthy and malignant thymocytes. As such, our studies collectively support the use of CK2 inhibitors (e.g. CX-4945) as putative therapy for both αβ and γδ T-ALL.
